# Agromorphologic, genetic and methylation profiling of *Dioscorea* and *Musa* species multiplied under three micropropagation systems

**DOI:** 10.1371/journal.pone.0216717

**Published:** 2019-05-16

**Authors:** Temitope Jekayinoluwa, Badara Gueye, Ranjana Bhattacharjee, Oladele Osibanjo, Trushar Shah, Michael Abberton

**Affiliations:** 1 International Institute of Tropical Agriculture, Ibadan, Oyo State, Nigeria; 2 Department of Chemistry, University of Ibadan, Ibadan, Oyo State, Nigeria; 3 International Institute of Tropical Agriculture, Nairobi, Kenya; Louisiana State University College of Agriculture, UNITED STATES

## Abstract

Plant *in vitro* vegetative propagation using classical semi-solid culture medium is limited due to the low degree of automation, suboptimal nutrient availability and induced physiological stress which often reduce its efficiency. Temporary Immersion System (TIS) emerged as an innovative approach to optimize and eliminate the drawbacks associated with the conventional system of micropropagation. In this study, both *Dioscorea* and *Musa* spp. were subjected to conventional semi-solid culture media, complete immersion in shaking liquid culture media and TIS using RITA bioreactor. *In vitro* grown plantlets were screened for possible vegetative changes using agro-morphological descriptors while genetic and methylation differences were assessed using amplified fragment length polymorphism (AFLP) and methylation-sensitive amplification polymorphism (MSAP). *In vitro* results showed that the number of shoots produced in *Musa* spp. varied significantly (P≤0.001) with the type of culture system. The highest mean shoot produced was observed with TIS (28.40) and the least using semi-solid culture medium (1.13). For *Dioscorea* spp., there was no significant interaction between the hormone combination and the culture system. However, the lowest mean shoot value (1.55) was observed in the semi-solid culture medium. Genetic analysis via AFLP using 15 primer pair combinations revealed that the 3 culture systems maintained genetic variation for *Musa* and *Dioscorea* spp. under *in vitro* and field conditions. Results showed 99% and 91% of the total bands were polymorphic under *in vitro* and field conditions respectively for *Musa* and 100% polymorphism for *Dioscorea* under *in vitro* and field conditions. Methylation investigation via MSAP using 12 primer pair combinations showed 25% and 46% polymorphic methylated-sensitive loci, 100% and 78% of non-methylated loci of the total bands generated under *in vitro* and field conditions respectively. Unmethylated (HPA+/MSP+) levels were highest in TIS (0.0842) as compared to CI (0.0227) and SS (0.0161) while full methylation or absence of target (HPA-/MSP-) was lowest in TIS (0.5890) and highest in SS (0.7138). For *Dioscorea*, 52% and 53% methylated sensitive loci and 100% non-methylated loci were polymorphic under *in vitro* and field conditions respectively. Although *in vitro* plant tissue culture techniques led to methylation at some loci of both species, there were no observable changes in the phenotype of both crops under field conditions. This also confirmed that not all methylation events lead to phenotypic changes.

## Introduction

*In vitro* plant tissue culture is recognized as one of the most valuable biotechnology tools for rapid multiplication of disease-free and true-to-type genotypes. The technology is used extensively in clonally propagated horticultural, food and tree crops. There are, however some challenges related to formation of aberrant plantlets and low survival during acclimatization stages in the field [1–3]. In both *Dioscorea* and *Musa* spp., *in vitro* clonal propagation can be used either for large scale propagation or conservation. However, somaclonal variation in plant material under *in vitro* plant tissue culture is significantly influenced by DNA methylation changes, although the occurrence of such events is unclear [4–6].

Culture media is a crucial aspect of *in vitro* plant propagation. It determines its effectiveness and can be targeted for improvement. The response of plant tissue to *in vitro* culture medium depends on several factors including the genotype itself, the nutrient content of the culture medium, the source and physiological state of the explant and the physical culture conditions such as temperature, pH, photoperiod and aeration [7–9] of the culture systems. Semi-solid and liquid culture are some of the common culture media systems used for *in vitro* plant propagation [10]. Although these methods have certain advantages but there are limitations too. The major disadvantages are asphyxiation, hyperhydricity, induced stress on agitated cultures, explant blackening (oxidation), poor diffusion rate and sub-optimal nutrient uptake which may lead to severe physiological disorder [10].

The temporary immersion system (TIS) that involves automated system provides an optimal environment for plant tissue and organ *in vitro* cultures. The method emerged as an approach to scale up the conventional method of propagation [11]. Over the years, several TIS have been developed and successfully used in various plant *in vitro* systems [12–14]. In 1993, Center de Cooperation Internationale en Recherche Agronomique pour le Development (CIRAD) developed a new temporary immersion system named RITA (recipient á immersion temporaire automatique) which eliminated the limitations of previously developed bioreactors, thus promoting massive *in vitro* plant production.

The objective of this study was therefore to explore the efficiency of TIS (RITA bioreactor) on *Musa* and *Dioscorea* spp. with respect to its comparative advantage over other conventional culture systems and to assess probable genetic and methylation modifications in the regenerated plants using amplified fragment length polymorphism (AFLP) and methylation-sensitive amplified polymorphism (MSAP) markers.

## Materials and methods

The accessions of *Musa* and *Dioscorea* spp. (**Table 1**) were collected from the Genetic Resources Center (GRC) of the International Institute of Tropical Agriculture (IITA) Ibadan, Nigeria.

**Table 1:** List of accessions of Yam and *Musa* with genome information and cultivar names.

Accessions	Cultivar	Genome
TMb 19	IJAU LAGADA	AA
TMb 26	MALACCENSIS HOLOTYPE	AA
TMb 28	MONJET	AA
TMb 42	PISANG BERLIN	AA
TMp 59	AGBAGBA	AAB
TMp 100	ESSANG	AAB
TMp 82	KLUE ROI WEE	AAB
TDr 1228	*Dioscorea rotundata*	N/A

### *In vitro* culture

Shoot tips of seven accessions of *Musa* spp. **(Table 1)** were cultured on Murashige and Skoog (MS) mineral-based culture medium [15] supplemented with 4.0 mg/l 6-Benylamionopurine (BAP) and 0.18mg/l Indole Acetic Acid (IAA) [16] (**S1 Table**). While one accession of *Dioscorea rotundata* (TDr 1228) (yam) was selected and screened using four hormone combinations to check its multiplication rate **(S1 Table)**.

Initiation of multiple shoots for *Musa* spp. was enhanced by wounding the apical meristem during subculture, a technique described by Jarret *et al*. [17] and Gupta [18] which involves vertical cuts through the meristematic dome while keeping the base of the explant intact.

The culture systems used for both species were: semisolid culture medium in test tube (SS), complete immersion in liquid culture medium in baby food jar (CI) with shaking on a rotary shaker at 100 rpm, and temporary immersion system (TIS) in RITA bioreactor with immersion time of 15min every 2 hours for both crops.

Five replicates of each of the seven accessions of *Musa* and one accession of *Dioscorea* were cultured under *in vitro* conditions in the three culture systems (SS, CI, TIS). The cultures were kept in growth chamber at 26°C ± 2.0, 38 μmol/m^2^/s and 12-hour photoperiod for three and six weeks for *Musa* spp. and *Dioscorea rotundata* respectively. The number of shoots were determined by counting the number of shoots (for *Musa*) and number of nodes (for yam) per single plant for each of the three culture systems. For better rooting system development, MS mineral-based culture medium was supplemented with either 0.18 mg/l IAA or no auxin for 3 weeks.

### Agro-morphological characterization

The *in vitro* grown planting materials were acclimatized (using sterilized top soil and chicken manure in ratio 2:1 for *Musa* and Jiffy peat pellet for *Dioscorea*) in the screen-house. After 12 weeks, the established plants were transplanted to the field at IITA (Latitude 7.50338° Longitude 3.90427°, Altitude 248.00m) following usual agronomic management practices such as regular watering, weeding and mulching. Data of agro-morphological parameters were captured every month following the available descriptor list for both *Musa* and *Dioscorea* [19–20] using 33 and 83 traits respectively until the crop senesced completely. However, data on majority of the traits were recorded at harvest for *Musa* spp. while for *Dioscorea rotundata* was carried out through the entire growth period. Mini tubers obtained from yam, were planted the second year and characterization data were collected on selected traits (**S2 and S3 Tables**).

### Molecular characterization

a) Sample collection and DNA isolation

About 100 mg of young leaf samples of *Musa* (cigar-like leaf) and *Dioscorea* (first fully expanded) genotypes were collected both from field and *in vitro* grown plants and labeled accordingly in sterile eppendorf tubes containing steel beads and immersed in liquid nitrogen. The samples were immediately homogenized to fine powder using a Geno Grinder (Retch MM 200) for 2 mins at a frequency of about 25 Hz. Genomic DNA was extracted from the ground samples using DNeasy plant Mini Kit (QIAGEN, 69106) and modified Dellaporta protocol [21] with the addition of DIECA and ascorbic acid to inhibit phenoloxidase and other impurities. Ground yam samples were first washed with 1000 μl HEPES buffer (10ml of 0.1 M HEPES + 90mg L-ascorbic acid + 102 mg PVP + 200μl β-mercaptoethanol) to remove secondary metabolites prior to extraction procedure. The quality of the DNA was determined by 1% agarose gel electrophoresis while the quantity and purity were measured through absorbance ratio (240/280) using NanoDrop spectrophotometer (Thermo scientific Nanodrop 2000 spectrophotometer).

b) AFLP and MSAP analysis

The AFLP and MSAP analysis followed a modified version of Vos *et al*., Vroh-Bi *et al*. and Reyna-Lopez *et al*. [22–24]. The isoschizomer restriction enzyme pair *Hpa*II and *Msp*I was used for MSAP, which recognizes CCGG site with differential sensitivity to methylation at cytosine, while *Mse*I was used for AFLP. When the internal cytosine is fully (methylation on both DNA strand) or hemi (methylation on one DNA strand) methylated, *Msp*I recognizes and cleaves the motif but it cannot cleave an outer cytosine. However, *Hpa*II has the capacity to recognize and cleave outer cytosine. Profiles generated from *Msp*I and *Hpa*II isochizomeric pair not only provide events associated with inner and outer methylation but give a comprehensive picture of genetic and epigenetic variations linked to methylation. Accordingly, we generated MSAP profiles of *in vitro* and field grown plants of both *Musa* and *Dioscorea* spp. to understand the methylation pattern associated with different culture system used in the study.

A restriction digest of 250ng genomic DNA (5 μl) with 20U/μl EcoRI (0.25μl), 10U/μl *Mse*I/*Msp*I/*Hpa*II (0.5μl), 10 X buffer 4 (5.0μl), 100X BSA (0.5μl) and ultra-pure molecular water was carried out in a thermo cycler for 3 hours at 37°C and the enzymes were inactivated *(Eco*RI/*Mse*I, *Eco*RI/*Msp*I *and Eco*RI/*Hpa*II) at 70°C for 15 minutes. This was followed by addition of 10μl of freshly prepared ligated mixtures [5 pmol *Eco*RI adapter (1.0 μl) + 50 pmol *Msp*I/*Mse*I adapter (1.0 μl) + 10X T_4_ Ligase buffer (1.0μl) + 100X BSA (0.5 μl) + T_4_ Ligase (0.5 μl) + ultra-pure molecular water (6.0 μl] to the digested sample to make a total of 50 μl reaction. The incubation was continued with a ligation process at 22°C for 5 hours, 70°C for 15 minutes and kept until further use. The ligated DNA fragment (2 μl) was used as template for pre-amplification in a thermocycler using the following composition and program: 10mM dNTP mix (0.5 μl), 25mM MgCl_2_ (0.6 μl), 10X standard Taq buffer (1.0 μl), 100 mg/μl BSA (1.0 μl), 5U/μl Taq polymerase (0.2 μl), 10nmol AFLP preselected primer pair (*Eco*RI/MseI, *Eco*RI/MspI) (1.0 μl), ligated DNA fragment (2.0 μl), ultra-pure water; 72°C for 2 minutes, 20 cycles at 94°C for 20 seconds, 56°C for 30 seconds and 72°C for 2 minutes, 4°C for ∞. The pre-amplification PCR product was then diluted with ultra-pure water in ratio of 1:10. The diluted pre-amplification product was further used as a template for the selective amplification PCR with a reaction volume of 10 μl comprising of 2.0 μl diluted pre-amplified DNA, 10 nmol/μl *Mse*I/*Msp*I/*Hpa*II primer (0.6 μl), 10nmol/ μl *Eco*RI primer (0.5 μl), 10mM dNTP mix (0.2 μl), 25mM MgCl_2_ (0.6 μl), 10X Taq buffer (1.0 μl), 5U/μl Taq polymerase (0.125 μl) and ultra-pure molecular grade water. The PCR program used for amplification is as follows: 95°C for 3 minutes, 36 cycles of 95°C for 30 seconds, 56°C for 1 minute, and 72°C for 1 minute and final extension at 72°C for 2 minutes. The PCR product (3 μl) and 7 μl of internal standard mix (HIDI and Liz) were vortexed, centrifuged and denatured for 5 minutes at 95°C prior to size fractioning in a capillary electrophoresis on ABI 3730. GeneScan 500 LIZ (applied Biosystems) was used as a size standard and POP 5 polymer (Applied Biosystems) was used for fluorescent labeling.

c) Scoring

The fragment peaks and intensity from the AFLP and MSAP analysis were evaluated using GeneScan software (Applied Biosystems) after scanning the signals from all samples for each crop separately. Following fragment analysis on ABI3730, AFLP and MSAP profiles were visualized using GeneMapper software v4 (Applied Biosystems, Foster City, CA, USA). Raw data generated were scored following a band-based strategy described by Bonin *et al*. [25] using the GeneMapper v4.0 software (applied Biosystems). Allelic profile was transformed into binary matrixes which were scored as ‘1’ for presence of allele and ‘0’ for absence of allele. In order to reduce eventual impact of size homoplasy [26], binning of allelic sizes was followed with a size range between 100–500 bp with peak height ≥100. All reactions were repeated twice and only distinct, polymorphic and informative bands across all samples were considered for analysis. Fragments that could not be visually distinguished with low intensity were regarded as ambiguous and were not scored.

### Statistical analysis

Phenotypic data generated from field grown plants were subjected to generalized linear model (PROC GLM and PROC GENMOD) in Statistical Analysis System (SAS-V9.2 and V9.3) [27] to obtain the variance components. The least significant mean (LSMEANS) was used to compare the means of different traits across both species. Principal coordinate analysis (PCoA) was carried out on standardized morphological data, eigen values and eigen vectors were calculated to generate two-dimensional plots under different growth conditions (*in vitro* and field). Similarly, genotypic (presence/absence) data was analyzed with *msap* software using R script (*msap_score*.*r*) [28–29] and GenAlEx version 6.5 [30]. Different statistical parameters such as PCoA, population differentiation test using analysis of molecular variance were also estimated using the Shannon diversity index.

## Results

### *In vitro* performance of genotypes in different culture systems

**Fig 1:**
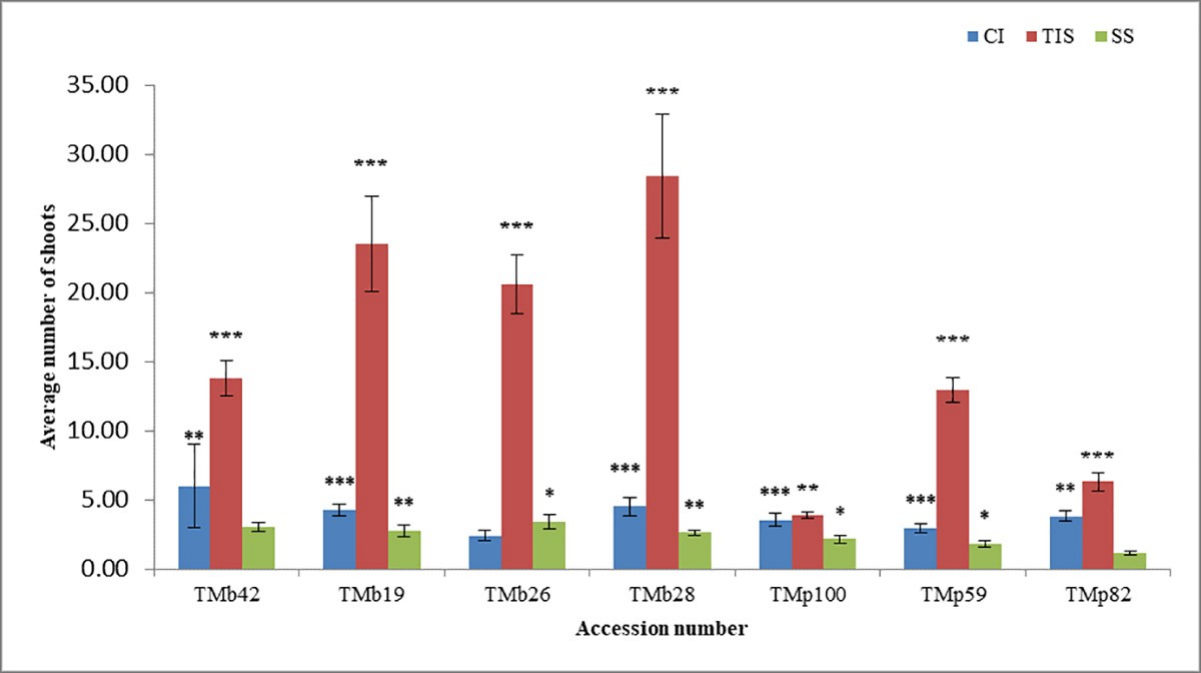
Effect of culture systems on *Musa* spp. under *in vitro* conditions. CI: Complete Immersion. TIS: Temporary Immersion System. SS: Semi-Solid.

The efficiency of the three culture systems used in the study were examined on two tropical crop species (*Musa* spp. and *Dioscorea rotundata*) after three and six weeks respectively. The number of shoots per explant were counted for each plant cultured on these culture systems. For *Musa* spp. the multiplication rate varied significantly both with the type of culture system and the genotype tested. However, TIS (RITA bioreactor) gave significantly higher shoot mean across the genotypes, followed by complete immersion in liquid media system with shaking while the least was observed with semi-solid medium (**Fig 1**). There was a significant interaction between the performance of the accessions and culture system (**Table 2**). On average, a higher multiplication rate was observed with genotypes having AA genome as compared to those with AAB genome. In terms of number of shoots produced under TIS (RITA bioreactor), the highest mean shoot of 28.40 was observed in TMb 28, while least mean shoot value of 3.90 was observed for TMp 100 in RITA bioreactor.

**Table 2:** ANOVA summary for *in vitro Musa* spp. and *Dioscorea rotundata*.

*Musa*						
Source of variation	df	Mean square	Treatment	NBS LSMEAN		
			CI	3.92***		
Accession	6	324.96***	TIS	15.63***		
Treatment	2	2799.27***	SS	2.40***		
Accession*Treatment	12	221.53***				
Mean		6.04				
Error		15.90				
CV		66.01				
*Dioscorea rotundata*						
Source of Variation	df	Mean Square			LSMean	
		NBS	NBC		NBS	NBC
System	2	4.19**	19.55***	TIS	2.45***	2.88***
Treatment	3	0.89ns	0.69ns	CI	2.20***	3.62***
Treatment*System	6	0.42ns	0.54ns	SS	1.55***	1.55***
Mean		2.05	2.59			
Error		0.82	0.97			
CV		44.07	38.13			

NBS, Number of shoot; NBC, Number of nodal cutting; TIS, Temporary Immersion System; CI, Complete Immersion; SS, Semi-solid.

*, **, *** p values significance at 0.05, 0.01 and 0.001 respectively; ns, not significant

**Fig 2:**
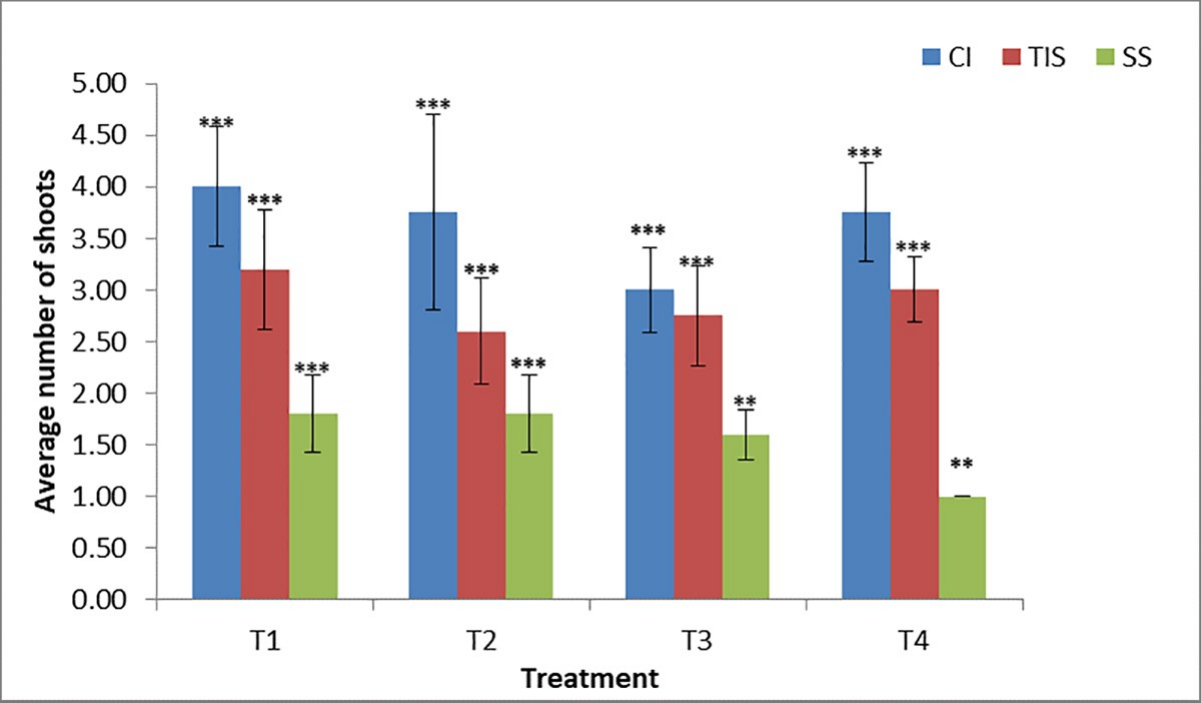
Treatment and culture system effect on *in vitro Dioscorea rotundata*. CI, Complete Immersion; TIS, Temporary Immersion System; SS, Semi-Solid.

For yam (*Dioscorea rotundata*), the result indicated that there was no significant difference in performance between TIS and CI within 6 weeks of plant culture while they differed significantly to SS across all the treatments used in the study (**Table 2**). Similarly, TIS and CI favored better multiplication rate than SS culture system (**Fig 2**). Culture media supplemented with low sucrose without hormone or with low level of Kinetin (T1, T2 & T4) promoted better shoot growth in TIS and CI. However, a previous study indicated that an increase in the culture duration in T1 and T4 favored shoot vigour in TIS (Jekayinoluwa *et al*. Unpublished, **S4 Table**).

### Agro morphological characterization

a) Screen house acclimatization

The *in vitro* plantlets were transferred to the screen-house for acclimatization and no significant difference was observed for the parameters (NL: Number of leaves, PH: plant height, LW: leaf width, LL: leaf length) measured (**S5 Table**) between the different culture systems for *Musa* spp.

There was significant difference in growth parameters of *Dioscorea* spp. when transferred to screen-house across the plants generated from different culture systems. (**S5 Table**). TIS showed better acclimatization success rate which differed significantly in terms of leaf width and plant height in comparison to other culture systems under treatment 4 (T4). However, T4 in CI produced higher number of leaves compared to plants cultured in other systems (**S5 Table**).

b) Field morphological characterization

The acclimatized plantlets of both *Musa* and *Dioscorea* accessions were transplanted under field conditions for morphological characterization. The *Musa* plants were screened based on thirty-three agro-morphological parameters. Only 1 (Weight of Bunch (WB)) variable out of 33 agro-morphological descriptors showed significant differences between the plants derived using the three culture systems. TIS recorded the highest value for WB in comparison to SS and CI (**S6A & S6B Table)** for TMb 19, TMp 59 and TMp 100. While for qualitative variables, the p-value of the Chi-square distribution showed no significant difference across the three culture systems.

For *Dioscorea*, about 8% of the total (85) agro-morphological variables showed a varying level of differences across the culture systems used. However, six qualitative traits such as leaf density, plant vigour, spine length, number of inflorescence, tuber shape and place of root on tuber were observed on plants cultured on TIS (**S7A–S7E Table).**

c) Molecular characterization

**Fig 3:**
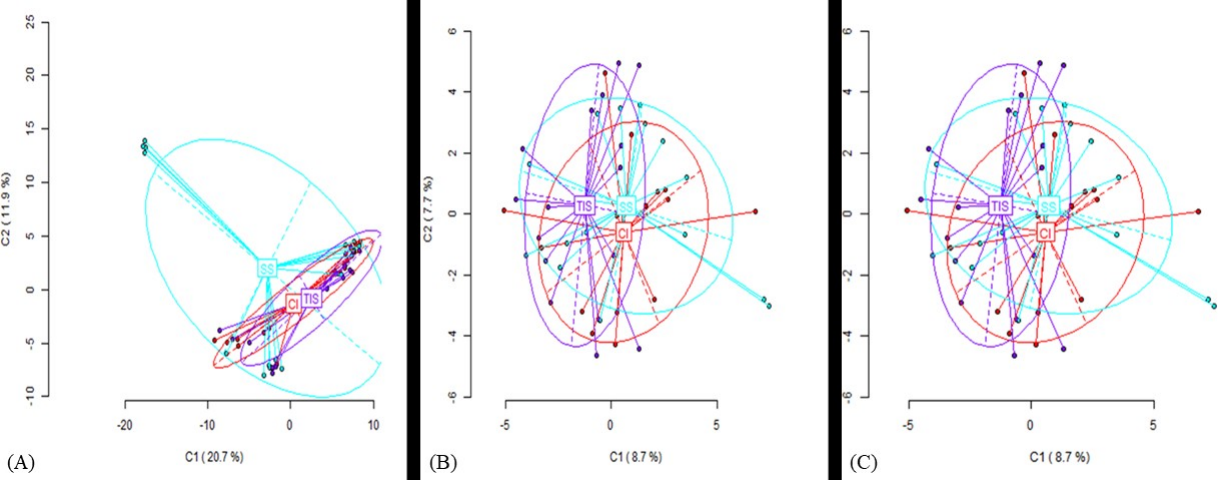
PCoA for *Musa* plants. (A) PCoA *Mse*I for *Musa* field plants. CI, Complete Immersion; TIS, Temporary Immersion System; SS, Semi-Solid, (B) PCoA on MSL for *Musa* field plants CI: Complete Immersion, TIS: Temporary Immersion System, SS: Semi-Solid, (C) PCoA on NML for *Musa* field plants CI: Complete Immersion, TIS: Temporary Immersion System, SS: Semi-Solid.

**Fig 4:**
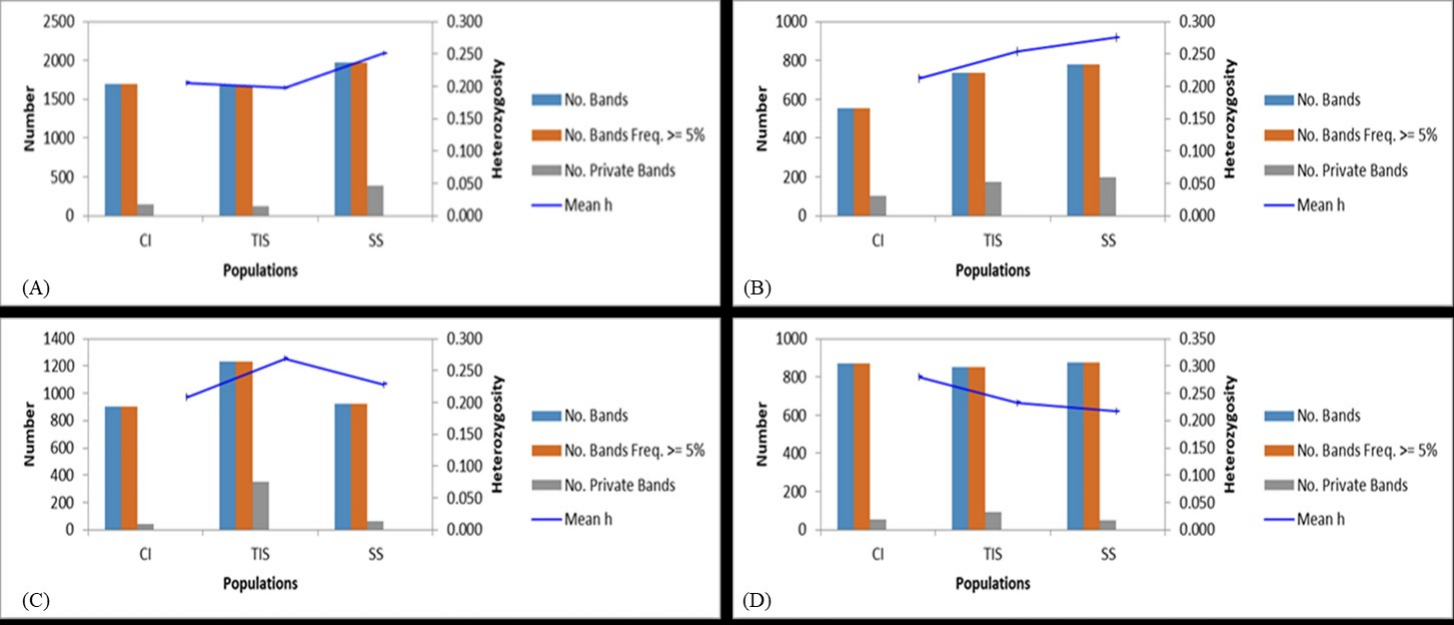
Band Patterns for *Musa* and Yam. (A) Band patterns across populations for *Musa* under field conditions (*Mse*I). CI, Complete Immersion; TIS, Temporary Immersion System; SS, Semi-Solid, (B) Band patterns across populations for *Musa* under *in vitro* conditions (*Mse*I). CI, Complete Immersion; TIS, Temporary Immersion System; SS, Semi-Solid, (C) Band patterns across populations for Yam under field conditions (*Mse*I). CI, Complete Immersion; TIS, Temporary Immersion System; SS, Semi-Solid, (D) Band patterns across populations for Yam under *in vitro* conditions (*Mse*I). CI, Complete Immersion; TIS, Temporary Immersion System; SS, Semi-Solid.

Amplified fragment length polymorphism (AFLP) profiles were generated for *in vitro* and field-grown plants of *Musa* and *Dioscorea* spp. with respect to different culture systems and treatments (**Table 3**). The profiles recorded 99–100% polymorphism regardless of the type of culture systems in both crops indicating the polymorphic nature of the markers used in the study. Principal Coordinate Analysis (PCoA) revealed the relatedness of the culture systems across the genotype used (**Fig 3**). The banding pattern for *Musa* spp. under field conditions showed clearly that TIS and CI have similar number of private bands (unique alleles) while SS recorded the highest with 387 private bands. Under *in vitro* conditions, SS recorded the highest number of private bands (200) while CI had the least number of private bands (103). Similarly, in *Dioscorea* spp., TIS derived field-grown plants recorded highest number (350) of private bands (**Fig 4**). The variance among the three culture systems (SS, CI & TIS) for *Musa* spp. under field condition was up to 3% (**S8 Table**) while there was no variation under *in vitro* conditions (**S8 Table**) indicating similar growth patterns among plantlets with negligible variances across different culture systems under study. Conversely, *Dioscorea* plants cultured under *in vitro* conditions recorded 17% variation among the different culture systems (**S8 Table**) while no variation was observed under field conditions (**S8 Table**).

**Table 3:** AFLP profile summary for *Musa* and Yam under *in vitro* and field conditions.

S/N	Sample Group	No. primer combination	No. Loci	polymorphic AFLP	% polymorphic AFLP	phi_ST (AFLP)	p_phi_ST (AFLP)
**1**	Musa *In vitro*	15	1115	1107	99	-0.02688	0.7383
**2**	Musa field	15	2566	2327	91	0.0255	0.0620
**3**	Yam *In vitro*	15	1050	1046	100	0.1670	0.0019
**4**	Yam field	15	1375	1375	100	0.001968	0.4227

**Fig 5:**
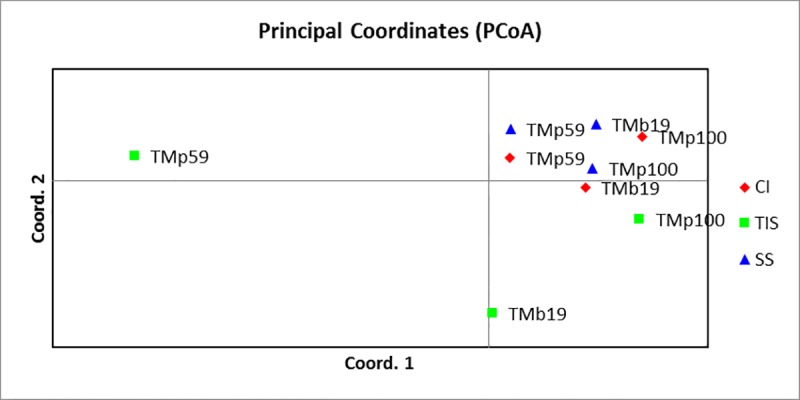
PCoA for *Musa in vitro* with *Hpa*II. CI: Complete Immersion, TIS: Temporary Immersion System, SS: Semi-Solid.

Methylation event was revealed by the PCoA analysis developed from binary matrix of combined MSAP profiles using *Eco*RI/*Msp*I and *Eco*RI/*Hpa*II. For *Musa* spp., the profile (**Table 4**) revealed similarity in both methylation susceptible loci (MSL) and non-methylated loci (NML) for plants grown using all the three culture systems, and in the field (**Fig 3B and 3C**). There were 564 (46% of total MSL) polymorphic methylated susceptible loci (MSL) and 797 (78% of total NML) non-methylated loci (NML) for field grown *Musa* plants. On the other hand, *in vitro* plants recorded 137 (25% of total MSL) polymorphic methylated susceptible loci and 15 polymorphic NML. The profiles of isoschizomer pair *Hpa*II, (^m^ CCGG) for field grown plants showed a variation of 1% among the culture systems (**S8 Table**) and CI culture system recorded the highest number of private bands (143) while SS had the least (100 bands). For *Msp*I (C^m^CGG), there was no observable variation among the three culture systems. However, samples collected from SS culture system recorded the highest private bands (141) while those from CI showed the least with 100 private bands. On the other hand, SS-derived plants recorded the least private bands in both profiles of the *HpaII* and MspI isoschizomer for *in vitro* grown *Musa* plants. The PCoA of *Hpa*II (^m^ CCGG) isoschizomer showed clustering of SS and CI grown plantlets together regardless of the genotype whereas, TIS grown plantlets are separated (**Fig 5**).

**Table 4:** MSAP profile summary for *Musa* and Yam.

Sample Group	No. Loci	NML	MSL	PNML (%)	PMSL (%)	p. wilcoxon	p_PhiST_MSL	p_PhiST_NML	SIMSL	SDMSL	SINML	SDNML
Musa *In vitro*	573	15	558	25	100	<0.0001	0.9897	1	0.5799	1.7859	0.3488	1.4174
Musa field	2246	1019	1227	78	46	<0.0001	0.3518	0.1567	0.4675	1.5960	0.1744	1.1905
Yam *In vitro*_CS	1563	367	1196	100	52	<0.0001	0.4597	3.00E-04	0.5910	1.8058	0.3518	1.4216
Yam field_CS	1993	563	1430	100	53	<0.0001	0.7763	0.4461	0.5231	1.6872	0.2044	1.2268

NML, non-methylated loci; MSL, methylated sensitive loci; PNML, polymorphic non-methylated loci; PMSL, polymorphic methylated sensitive loci; SIMSL, Shannon Index_MSL; SDMSL, Shannon diversity_MSL; SINML, Shannon Index_NML; SDNML, Shannon diversity_NML

**Fig 6:**
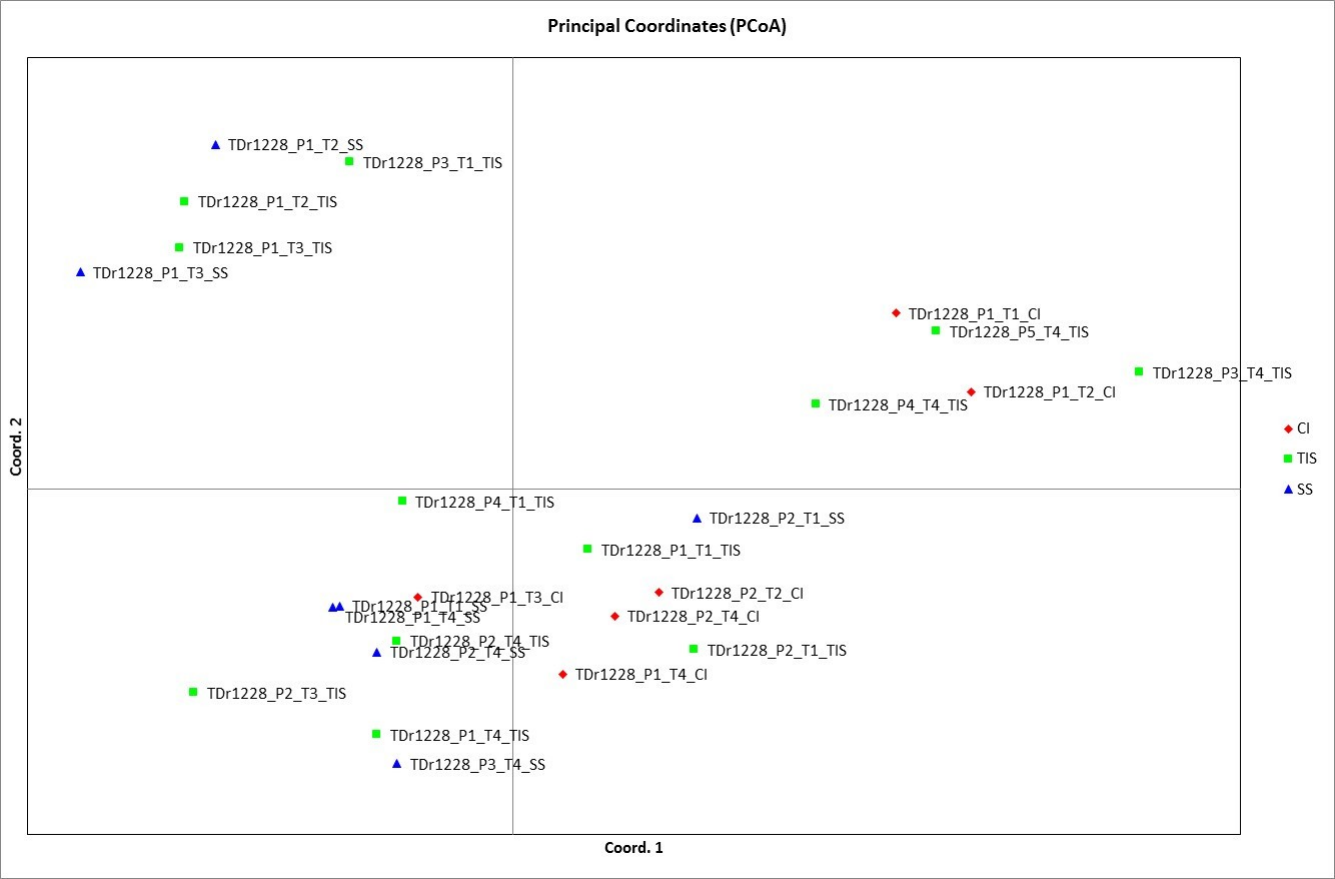
PCoA for Yam in field with *Hpa*II. CI: Complete Immersion, TIS: Temporary Immersion System, SS: Semi-Solid.

Twenty-five field-grown yam plants of TDr 1228 accession generated 757 polymorphic MSL bands and 563 polymorphic NML bands. The PCoA generated from *Hpa*II profile, showed clustering based on different treatments used in culture system (**Fig 6**). T1 and T4 were closely related while T2 and T3 clustered together. There were 627 polymorphic MSL and 367 polymorphic NML for *in vitro* grown plants. Further clustering based on culture system in relation to the treatment used revealed TIS with minimum MSL across all treatments. Of all the treatments, T1 and T4 recorded 334 and 338 MSL while 311 and 270 NML, respectively (**Table 5**). The measure of genetic diversity was estimated by the Shannon diversity index and comparison with Wilcoxon test revealed a significant difference (P<0.0001) between MSL and NML for both crops.

**Table 5:** Summary of band pattern of *in vitro Dioscorea rotundata*.

S/N	Accession	Treatment	Culture system	MSL	NML
1	TDr 1228	T1	CI	518	231
2	TDr 1228	T1	TIS	334	311
3	TDr 1228	T1	SS	542	271
4	TDr 1228	T2	CI	468	129
5	TDr 1228	T2	TIS	366	130
6	TDr 1228	T2	SS	573	258
7	TDr 1228	T3	CI	397	176
8	TDr 1228	T3	TIS	380	242
9	TDr 1228	T3	SS	577	243
10	TDr 1228	T4	CI	375	197
11	TDr 1228	T4	TIS	338	270
12	TDr 1228	T4	SS	572	260

MSL: Methylation sensitive loci, NML: Non-methylation loci, CI: Complete Immersion, TIS: Temporary Immersion System, SS: Semi-Solid

## Discussion

In this study, three *in vitro* culture systems were used, including semisolid medium in test tube, complete immersion in liquid culture medium with shaking in baby food jar and temporary immersion system in RITA bioreactor for *Musa* and *Dioscorea* spp. It was noted that the type of culture system had a significant effect on the number of polyshoot produced *in vitro*. In general, the temporary immersion system was more efficient for rapid propagation of *Musa* spp., owing to the higher number of shoots produced within a relatively short period of time compared to other conventional (semi-solid) methods. An optimal relatively short period of 21 days generated a multiplication rate of 28.40 and 12.93 for *Musa* AA and AAB respectively in TIS (RITA bioreactor). This can enable a faster rate of availability of plantlets for use and conservation. Similar results were observed by Roels *et al*. [31] at 28 days with multiplication rate of 13 and Hui *et al*. [32] at 5 weeks with an average rate of 4.9. The studies of Businge *et al*. [33], Georgieva *et al*., [34] and Steinmacher *et al*., [35] also confirmed the positive effect of TIS over conventional *in vitro* propagation in other crops. This may be because TIS has the advantage of allowing better contact between the explant and the culture medium, thus easy diffusion and uptake of nutrient is achieved. The TIS nullifies the drawbacks of liquid culture medium by making the explant immersed only for a while and aerating them inducing relatively less stress on the tissues [36]. Conversely, there was no significant effect of culture system on the multiplication rate of *Dioscorea* spp. TDr 1228 within 6 weeks of culture. However, the lowest multiplication rate was observed in semi-solid culture medium followed by TIS and CI. This suggested that an increased nutrient-to-plant contact period was needed for increased multiplication rate of TDr 1228. Also, culture media composition influenced the multiplication rate of TDr 1228. The multiplication rate was highest in MS medium supplemented with 0.5mg/l Kinetin (T1) in CI, TIS and SS. While the absence of hormone supplement (T4) also supported growth in CI and TIS but minimal in SS. Polizin *et al*. [37], observed a similar trend, wherein there was no significant difference between TIS and SS at 8 weeks and suggested increasing immersion frequency as a possible way of optimizing the potential of TIS for *Dioscorea* spp. However, Yan *et al*. [38], observed significant difference in the growth rate of *Dioscorea fordii* and *Dioscorea alata* in TIS indicating that the multiplication rate in *Dioscorea* spp. may be genotype/cultivar dependent.

TIS is also known to improve plant quality and increase shoot vigor as well as quantity of morphologically normal somatic embryos [39]. Hyperhydricity that seriously affects cultures in liquid medium is eliminated in TIS [10] as the explants are not permanently immersed. Hvoself-Eide *et al*., [40] confirmed that in TIS, there is increased multiplication rate when shoots are appropriately exposed to culture media at correct intervals. TIS provides an excellent way of using liquid medium at the same time controlling the gaseous environment thereby increasing the growth and multiplication rate of cultures. Also due to lack of agitation, the mechanical stress on plant tissues are low compared to other micropropagation methods.

For *in vitro* plant tissue culture, type of growth regulator plays an important role for the physiological response of explants. A higher level of BAP supplement in MS medium promoted the production of polyshoot for *Musa* spp. As reported by several authors [41–44], polyshoot production in *Musa* spp. was improved by wounding the apical meristem to break the apical dominance thereby stimulating the axillary buds to produce multiple shoots in *Musa*. For *Dioscorea rotundata*, low sucrose concentration has been identified to reduce exudation of phenolic compounds, which may hamper regeneration and growth [45]. This in combination with no or low levels of plant hormone have been identified to promote its multiplication rate [46–48].

Molecular characterization of plants obtained from the three culture systems was essential to compare their genetic differences in relation to phenotypic characteristics. Amplified Fragment Length Polymorphism (AFLP) and Methylation Sensitive Amplification Polymorphism (MSAP) are useful molecular markers that help to understand effect of methylation on genetic diversity within and among a population. This is possible because AFLP marker is highly polymorphic and evenly distributed in the genome, giving a broad understanding of genomic variation. MSAP is a modification of AFLP marker that reveals methylation pattern in a population.

AFLP profiles for *Dioscorea* and *Musa* spp. under *in vitro* and subsequent field conditions revealed a level of conserved genetic variability across the genotypes. A 3% variation among the different culture systems was explained on the basis of presence of private alleles peculiar to each type of culture system. In addition, the pairwise genetic distances were calculated to investigate the allelic differences among the 3 culture systems. A low genetic distance was observed between SS and TIS for both *Dioscorea* and *Musa spp*. either under *in vitro* or field conditions, indicating the relatedness between the two culture systems.

In this study, MSAP using *Eco*RI/*Hpa*II and *Eco*RI/*MSp*I as restriction enzymes to identify and cleave methylation regions, thus generating methylation profiles which can help in understanding genetic diversity between and among a population. In plant genome, DNA methylation is a common phenomenon that does not alter the main genetic code but may show somatic or phenotypic variations. Schulz et al [49] and Herrera and Bazaga [50] described different forms of cytosine methylation that explained the basic principle of methylation scoring and profiling. Cleavage of methylated cytosine by *Msp*I and *Hpa*II could either result in full methylation (when the internal cytosine of the double stranded DNA is methylated) or hemi-methylation (methylation of internal cytosine on one DNA strand) with the exception that *Hpa*II cleaves external cytosine. CG methylation is said to be an important factor for promoter function [51]. This is evident in the work of Hafiz *et al* [52] who made it clear that DNA methylation plays a significant role in the transition from vegetative to reproductive growth and ploidy level of plants. Polymorphism in DNA methylation is an important form of genetic variation which plays a significant role in cell division, higher growth rate of plants, rooting ability and a potential capacity of silencing plant viruses [53–54].

Methylation profiles generated from *Musa* and *Dioscorea spp*. under field and *in vitro* conditions revealed a significant level of full and partial methylation pattern. The value of Shannon diversity accounts for the richness and evenness of MSL and NML for *Musa* and *Dioscorea* spp. under field and *in vitro* conditions. The level of diversity of MSL under *in vitro* (1.78) condition-was higher than field (1.59) condition for *Musa* spp. A similar trend was observed for *Dioscorea* spp. The relative lower diversity value under field condition may be a reflection of environmental influence on the crops. It has been reported that factors such as plant growth hormone, increased level of salt, biotic or abiotic stress may contribute to methylation/genetic variation in crops. This was confirmed with the number of MSL for *Dioscorea* spp. across four hormone treatments and the 3 culture systems used under *in vitro* conditions. Plants grown in TIS recorded lowest number of MSL across all hormone treatment used while SS system had the highest number of MSL. A closer look at the hormone combination revealed that T1 and T4 had a lower number of MSL compared to other hormone combinations. LoSchiavo *et al* [55] and Arnholdt-Schmitt [56] reported hypo-methylation with increasing level of cytokinin (kinetin) in carrot root while higher concentration of auxin (2, 4-D) increased methylation level from 15 to 70%. This explained how plants react or adjust to stressful conditions when developing different cell types [57–58]. Rico *et al* [59] also confirmed increase in hemi-methylation level and decrease in full methylation of drought effect on forest trees. The DNA methylation highlights the capacity of plants to acclimatize and adapt to changing environmental conditions. An exponential of the Shannon diversity index provided information on the effective number of species, which is the actual measure of diversity as it shows the richness and evenness of a population [60–61].

## Conclusion

The present study confirmed the advantage of temporary immersion system (TIS, RITA bioreactor) in improving the multiplication rate of polyshoot production in both *Musa* and *Dioscorea* spp. The adoption of TIS over other propagation system is to assist in overcoming the challenges of mass production of good quality planting materials within a relatively short period of time and at a lower cost. The suitability of tissue culture-based system depends on their effect on genetic uniformity. Methylation-sensitive amplification polymorphism (MSAP) is a valuable tool for detecting methylation, which could be a potential indicative signal of possible somaclonal variation in clonal crops with respect to the culture duration and/or systems. The culture systems used in this study did not show significant alteration on the genetic integrity of *Musa* and *Dioscorea* spp. The high level of genetic polymorphism showed the ability of the culture system to conserve crop genetic variability, which can make the crop adaptable and promote their use and conservation in genebanks, breeding and biotechnology programs. However, factors such as plant growth hormone, culture system type, mode of propagation and induced stress revealed the cause of variation in plants. It was also observed that certain type of plant growth hormone could either trigger increase or decrease in methylation, which could lead to activation or deactivation of certain genes in the plant genome. The variation observed is marked by increase or decrease in methylation events and could be further explored to understand and assess epigenetic changes in these two plants.

## Supporting information

S1 Table *In vitro* Culture medium composition. MSCM, multiple shoot culture medium; RCM, rooting culture medium; T1, Treatment 1; T2, Treatment 2; T3, Treatment 3; T4, Treatment 4.
(DOC)

S2 Table Agro-morphological descriptors for *Musa* spp. (DOC)

S3 Table Agro-morphological descriptors for *Dioscorea* spp. (DOC)

S4 Table ANOVA summary for *in vitro* yam at 6 and 23 weeks. (DOC)

S5 Table ANOVA summary for *Musa*&Dioscorea_screenhouse, NL, Number of leaves; PH, plant height; LW, leaf width; LL, leaf length; CS, Culture system, *, **, ***, p values significance at 0.05, 0.01 and 0.001 respectively; ns, not significant. (DOC)

S6 Table Frequency&ANOVA summary for Musa traits. *, **, ***, p values significance at 0.05, 0.01 and 0.001 respectively; WB, weight of bunch; NHWB, Number of hands on whole bunch; NFTH, Number of fruit on third hand; FL, Fruit length; NFD, Number of days to flowering; TIS, Temporary Immersion System; SS, Semi-Solid; CI, Complete Immersion.
(DOC)

S7 Table Frequency&ANOVA summary for Yam traits. DOH, Date of harvest; NTY1, Number of tuber year 1; NTY2, Number of Tuber year 2; WTY1, Weight of tuber year 1; WTY2, Weight of tuber year 2; LOT, Length of tuber; WOT, width of tuber; IL, internode length, DOFAE, days to flowering after emergence; MFL, male flower length; NOSPP, Number of stem per plant; NOI, Number of internode, *, **, ***, p values significance at 0.05, 0.01, 0.001 respectively; ns, not significant; TIS, temporary immersion system; SS, Semi-Solid, CI, complete immersion.
(DOC)

S8 Table AMOVA summary for *Musa* and *Dioscorea*. (A) AMOVA summary for *Musa* under field condition using MSeI restriction enzyme, (B) AMOVA summary for *Musa* under *in vitro* conditions using MSeI restriction enzyme, (C) AMOVA summary for *Dioscorea* under *in vitro* condition using MSeI restriction enzyme (D) AMOVA summary for *Dioscorea* under field condition using MSeI restriction enzyme (E) AMOVA summary for Musa under field condition using HpaII restriction enzyme. Df, degree of freedom; SS, sum of squares; MS, mean squares; Est. var, estimated variance.
(DOC)
