# Supplementary material for: Agromorphologic, genetic and methylation profiling of Dioscorea and Musa species multiplied under three micropropagation systems
Source: PLoS One. 2019 May 16;14(5):e0216717. doi: 10.1371/journal.pone.0216717 (PMC6522119; doi:10.1371/journal.pone.0216717)
Supplement: S1 Table — MSCM, multiple shoot culture medium; RCM, rooting culture medium; T1, Treatment 1; T2, Treatment 2; T3, Treatment 3; T4, Treatment 4. (DOC) [file pone.0216717.s001.doc]

**S1 Table: *In vitro* multiple shoot culture medium (MSCM) and rooting culture (RCM) medium composition for *Musa* spp. And *Dioscorea* spp.**

| ***Musa* spp.** | | | |  |
| --- | --- | --- | --- | --- |
| **Component** | **Quantity/l (MSCM)** | **Quantity/l (RCM)** |  |  |
| MS Salt | 4.43g | 4.43g |  |  |
| Sucrose | 30g | 30g |  |  |
| Ascorbic acid | 10mg | 10mg |  |  |
| BAP | 4mg | 0 |  |  |
| IAA | 0.18mg | 0.18mg (optional) |  |  |
| Gelrite | 2g (for Semi-Solid only) | 2g |  |  |
| ***Dioscorea* spp.** | | | | |
| **Component** | **T1** | **T2** | **T3** | **T4** |
| MS Salt (g) | 4.43 | 4.43 | 4.43 | 4.43 |
| Myo-inositol (mg) | 100 | 100 | 100 | 0 |
| Kinetin (mg) | 0.5 | 2 | 2 | 0 |
| NAA (mg) | 0 | 0.1 | 0 | 0 |
| Ascorbic acid (mg) | 0 | 0 | 0 | 0 |
| Sucrose (g) | 30 | 30 | 30 | 20 |
| L-Cystein (mg) | 20 | 20 | 20 | 0 |
