# Supplementary material for: Agromorphologic, genetic and methylation profiling of Dioscorea and Musa species multiplied under three micropropagation systems
Source: PLoS One. 2019 May 16;14(5):e0216717. doi: 10.1371/journal.pone.0216717 (PMC6522119; doi:10.1371/journal.pone.0216717)
Supplement: S2 Table — (DOC) [file pone.0216717.s002.doc]

**S2 Table: Agro-morphological descriptors for *Musa*** spp.

| ***Musa* Descriptors** | **Acronym** |
| --- | --- |
| Weight of Bunch | WB |
| Number of hands on the whole bunch | NHWB |
| Number of fruits on third hand | NFTH |
| Fruit length | FL |
| Number of days to flowering | NFD |
| Pseudostem height | PH |
| Predominant underlying colour of the pseudostem | CP |
| Sap colour | SC |
| Blotches at petiole base | BPB |
| Petiole canal of the 3rd leaf | PC |
| Petiole margins | PM |
| Petiole margin colour | PMC |
| Edge of petiole margin | EPM |
| Colour of cigar leaf dorsal surface | CCLD |
| Bunch position | BP |
| Bunch shape | BS |
| Rachis position | RP |
| Rachis appearance | RA |
| Male bud shape | MBS |
| Bract apex shape | BAS |
| Bract imbrication | BI |
| Colour of the bract external face | CBE |
| Colour of bract internal face | CBI |
| Bract behaviour before falling | BBBF |
| Compound tepal basic colour | CTBC |
| Lobe colour of compound tepal | LCCT |
| Anther colour | AC |
| Dominant colour of male flower | DCMF |
| Fruit Shape | FS |
| Fruit apex | FA |
| Remains of flower relicts at fruit apex | RFR |
| Fruit pedicel length (mm) | FPL |
| Fusion of pedicels (before they join the crown) | FP |
