# Supplementary material for: Agromorphologic, genetic and methylation profiling of Dioscorea and Musa species multiplied under three micropropagation systems
Source: PLoS One. 2019 May 16;14(5):e0216717. doi: 10.1371/journal.pone.0216717 (PMC6522119; doi:10.1371/journal.pone.0216717)
Supplement: S3 Table — (DOC) [file pone.0216717.s003.doc]

**S3 Table: Agro-morphological descriptors for *Dioscorea*** spp.

| ***Dioscorea* Descriptors** | **Acronym** |
| --- | --- |
| Leaf colour | LC |
| Leaf margin colour | LMC |
| Vein colour | VC |
| Petiole colour | PC |
| Petiole wing colour | PWC |
| First leaf emergence | FLE |
| Stem colour | SC |
| Waxiness | WAX |
| ABSENCE/PRESENCE of wings | AP |
| Wing colour | WC |
| Absence/presence of colour of spine at spine base | CSSB |
| Absence/presence of spine | APS |
| Barky patches | BP |
| Position of leaves | PL |
| Leaf density | LD |
| Leaf type | LT |
| Leaf margin | LM |
| Leaf lobation | LL |
| Leaf vein colour (upper surface) | LVCU |
| Leaf vein colour (lower surface) | LVCL |
| Leaf margin colour | LMC |
| Leaf shape | LS |
| Leaf apex shape | LA |
| Undulation of leaf | UOL |
| Upward folding of leaf along main vein | UFLAMV |
| Downward arching of leaf along main vein | DALAMV |
| Upward folding of leaf lobes to form cup | UFLLFC |
| Position of the widest part of the leaf | PWPL |
| Tip length | TL |
| Tip colour | TC |
| Petiole length | PL2 |
| Petiole length in correlation to leaf blade | PLCLB |
| Petiole colour | PC |
| Plant type | PTH |
| Vigour | VIG |
| Twining habit | TH |
| Twining direction | TD |
| Stem height | SH |
| Stem colour | SC |
| Stem cross-section shape at base | SCSSB |
| Spine on stem base | SSB |
| Spine on stem above base | SSAB |
| Spine position | SP |
| Spine shape | SS |
| Spine length | SL |
| Sex | Sex |
| Inflorescence position | IP |
| No of inflorescence per plant | NIPP |
| Inflorescence type | IT |
| Average length of inflorescence | ALOF |
| Flower colour | FC |
| Relationship of tubers | ROT |
| Absence/presence of corms | APC |
| Corm ability to separate | CAS |
| Corm type | CT |
| Spininess of root | SOR |
| Sprout at harvest | SAH |
| Colour of sprout | COS |
| Tuber shape | TS |
| Tendency of tuber to branch | TTB |
| Place where tuber branch | PWTB |
| Spiny root on tuber surface | SRTS |
| Roots on tuber surface | ROTS |
| Place of root on tuber | POROT |
| Prickly appearance of the tuber | PAOTT |
| Wrinkles on tuber surface | WOTS |
| Absence/presence of cracks | APOC |
| Flesh colour upper | FCU |
| Flesh colour middle | FCM |
| Flesh colour lower | FCL |
| Tuber flesh texture | TFT |
| Flesh oxidation colour | FOC |
| Tuber skin colour beneath | TSCB |
| Date of harvest | DOH |
| Number of tuber year 1 | NTY1 |
| Weight of tuber year 1 | WTY1 |
| Number of tuber year 2 | NTY2 |
| Weight of tuber year 2 | WTY2 |
| Length of tuber | LOT |
| Width of tuber | WOT |
| Internode length | IL |
| Days to flowering after emergence | DOFAE |
| Male flower length | MFL |
| No of internode | NOI |
| No of stems per plant | NOSPP |
