# Supplementary material for: Agromorphologic, genetic and methylation profiling of Dioscorea and Musa species multiplied under three micropropagation systems
Source: PLoS One. 2019 May 16;14(5):e0216717. doi: 10.1371/journal.pone.0216717 (PMC6522119; doi:10.1371/journal.pone.0216717)
Supplement: S4 Table — (DOC) [file pone.0216717.s004.doc]

**S4 TABLE: ANOVA summary for *in vitro* yam at 6 and 23 weeks in culture**

| Source of Variation | df | Mean Square |  |  | LSMean | |
| --- | --- | --- | --- | --- | --- | --- |
|  |  | NBS | NBC |  | NBS | NBC |
| Duration | 1 | 2.45* | 1940.45*** | 6Weeks | 2.70*** | 3.10* |
| Treatment | 1 | 0.45ns | 22.05ns | 23Weeks | 2.00*** | 22.80*** |
| Treatment*Duration | 1 | 0.45ns | 18.05ns |  |  |  |
| Mean |  | 2.35 | 12.95 |  |  |  |
| Error |  | 0.45 | 20.9 |  |  |  |
| CV |  | 28.54 | 35.3 |  |  |  |

NBS, Number of shoot; NBC, Number of nodal cuttings; ns, not significant; *, ***, p value significance at 0.05 and 0.001 respectively
