# Supplementary material for: Agromorphologic, genetic and methylation profiling of Dioscorea and Musa species multiplied under three micropropagation systems
Source: PLoS One. 2019 May 16;14(5):e0216717. doi: 10.1371/journal.pone.0216717 (PMC6522119; doi:10.1371/journal.pone.0216717)
Supplement: S5 Table — (DOC) [file pone.0216717.s005.doc]

**S5 Table: (A) ANOVA summary for *Musa* spp. under screen-house condition (B) ANOVA summary for *Dioscorea* spp. under screen-house condition.**

| (A)Mean square for *Musa* under Screen-house condition | | | | | | |  |  |
| --- | --- | --- | --- | --- | --- | --- | --- | --- |
| Source of variation | df | NL | PH | | LW | LL |  |  |
| Replicate | 4 | 1.16 ns | 156.32 ns | | 28.02* | 155.10* |  |  |
| Accession | 5 | 7.60*** | 67.33 ns | | 39.62** | 60.02 ns |  |  |
| Treatment | 2 | 3.10 ns | 106.81 ns | | 16.05 ns | 77.02 ns |  |  |
| Accession*Treatment | 7 | 0.93 ns | 112.17 ns | | 26.75** | 105.09 ns |  |  |
| Mean |  | 8.38 | 31.81 | | 6.61 | 18.21 |  |  |
| Error |  | 1.3 | 74.5 | | 10.91 | 54.52 |  |  |
| CV |  | 13.62 | 27.13 | | 49.93 | 40.54 |  |  |
|  |  |  |  |  | |  |  |  |
| (B)Mean square for *Dioscorea* under Screen-house condition | | | | | |  |  |  |
| Source of variation | df | NL | PH | | LW |  |  |  |
| Replicate | 4 | 8.4 ns | 5.89 ns | | 1.26* |  |  |  |
| CS | 2 | 132.49*** | 60.72** | | 8.19*** |  |  |  |
| Treatment | 3 | 16.03 ns | 35.56** | | 6.25*** |  |  |  |
| CS*Treatment | 6 | 5.69 ns | 12.25 ns | | 1.47** |  |  |  |
| Mean |  | 7.52 | 5.09 | | 1.19 |  |  |  |
| Error |  | 14.14 | 6.78 | | 0.43 |  |  |  |
| CV |  | 49.98 | 51.13 | | 55.49 |  |  |  |

NL, Number of leaves; PH, plant height; LW, leaf width; LL, leaf length; CS, Culture system, *, **, ***, p values significance at 0.05, 0.01 and 0.001 respectively; ns, not significant
