# Supplementary material for: Agromorphologic, genetic and methylation profiling of Dioscorea and Musa species multiplied under three micropropagation systems
Source: PLoS One. 2019 May 16;14(5):e0216717. doi: 10.1371/journal.pone.0216717 (PMC6522119; doi:10.1371/journal.pone.0216717)
Supplement: S6 Table — *, **, ***, p values significance at 0.05, 0.01 and 0.001 respectively; WB, weight of bunch; NHWB, Number of hands on whole bunch; NFTH, Number of fruit on third hand; FL, Fruit length; NFD, Number of days to flowering; TIS, Temporary Immersion System; SS, Semi-Solid; CI, Complete Immersion. (DOC) [file pone.0216717.s006.doc]

**S6a Table: Frequency table for *Musa* qualitative agro-morphological traits**

**
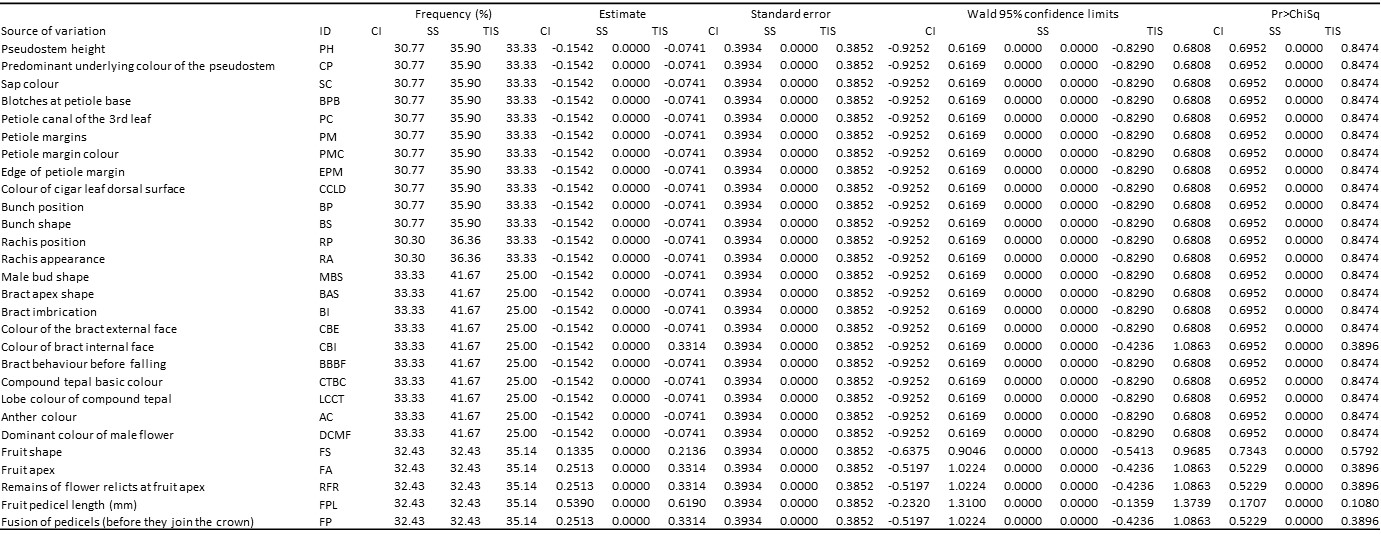
**

**S6b Table: ANOVA summary for *Musa* quantitative traits**

| **Source of Variation** | **df** | **Mean Square** | | | | | | |  | **LS Mean** | | | | |
| --- | --- | --- | --- | --- | --- | --- | --- | --- | --- | --- | --- | --- | --- | --- |
|  |  | **WB** | **NHWB** | **NFTH** | **FL** | **NFD** | **System** | **WB** | | | **NHWB** | **NFTH** | **FL** | **NFD** |
| Accession | 3 | 16.07*** | 32.50*** | 330.38*** | 573.12*** | 1060.99ns | TIS | 6.84*** | | | 7.34*** | 10.09*** | 21.46*** | 275.4*** |
| System | 2 | 16.06** | 1.28ns | 2.27ns | 25.39ns | 2512.65ns | SS | 4.85*** | | | 6.72*** | 9.42*** | 18.46*** | 277.12*** |
| Accession*System | 6 | 6.23** | 1.54ns | 1.07ns | 7.43ns | 647.78ns | CI | 4.65*** | | | 6.75*** | 9.22*** | 19.95*** | 302.64*** |
| Mean |  | 5.22 | 7.16 | 10.97 | 18.14 | 285.3 |  |  | | |  |  |  |  |
| Error |  | 1.99 | 1.18 | 3.55 | 9.5 | 1250.46 |  | | |  |  |  |  |  |
| CV |  | 27.03 | 15.2 | 17.19 | 16.99 | 12.39 |  | | |  |  |  |  |  |

*, **, ***, p values significance at 0.05, 0.01 and 0.001 respectively; WB, weight of bunch; NHWB, Number of hands on whole bunch; NFTH, Number of fruit on third hand; FL, Fruit length; NFD, Number of days to flowering; TIS, Temporary Immersion System; SS, Semi-Solid; CI, Complete Immersion.
