# Supplementary material for: Agromorphologic, genetic and methylation profiling of Dioscorea and Musa species multiplied under three micropropagation systems
Source: PLoS One. 2019 May 16;14(5):e0216717. doi: 10.1371/journal.pone.0216717 (PMC6522119; doi:10.1371/journal.pone.0216717)
Supplement: S7 Table — DOH, Date of harvest; NTY1, Number of tuber year 1; NTY2, Number of Tuber year 2; WTY1, Weight of tuber year 1; WTY2, Weight of tuber year 2; LOT, Length of tuber; WOT, width of tuber; IL, internode length, DOFAE, days to flowering after emergence; MFL, male flower length; NOSPP, Number of stem per plant; NOI, Number of internode, *, **, ***, p values significance at 0.05, 0.01, 0.001 respectively; ns, not significant; TIS, temporary immersion system; SS, Semi-Solid, CI, complete immersion. (DOC) [file pone.0216717.s007.doc]

**S7a Table: Frequency table for Yam qualitative agro-morphological traits_ young leaf and young stem**

**
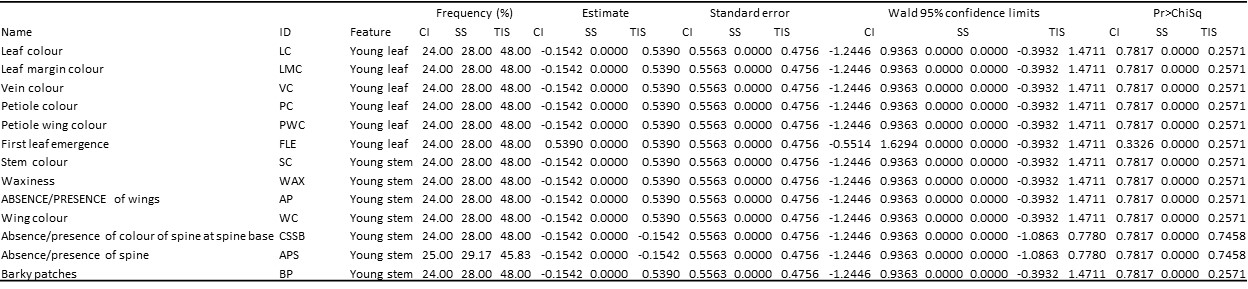
**

**S7b Table: Frequency table for Yam qualitative agro-morphological traits_ matured leaf**

**
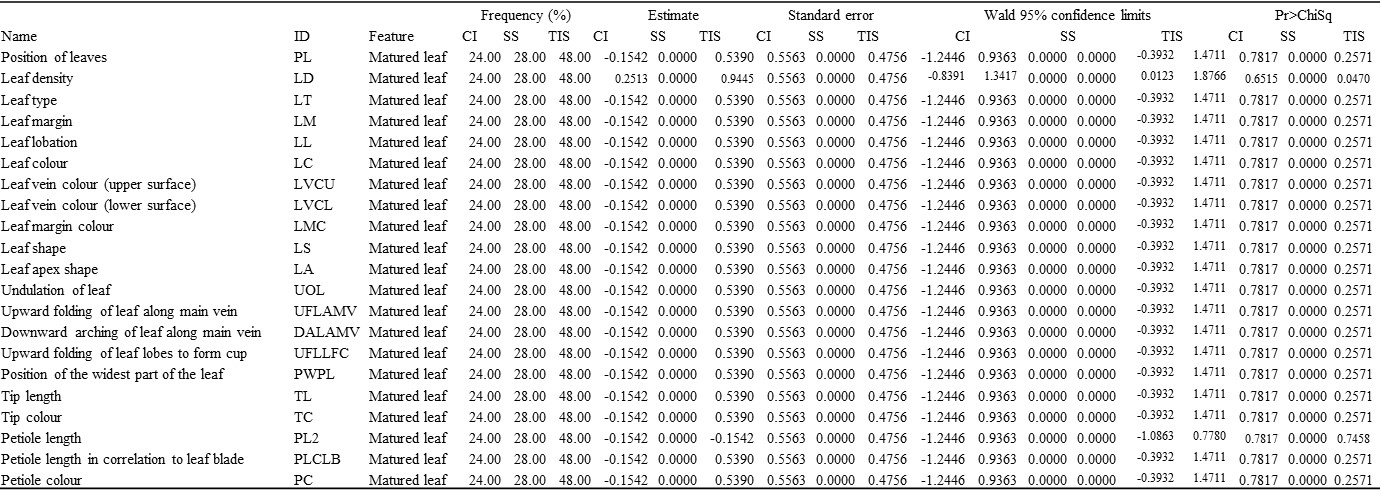
**

**S7c Table: Frequency table for Yam qualitative agro-morphological traits_ matured stem and flower**

**
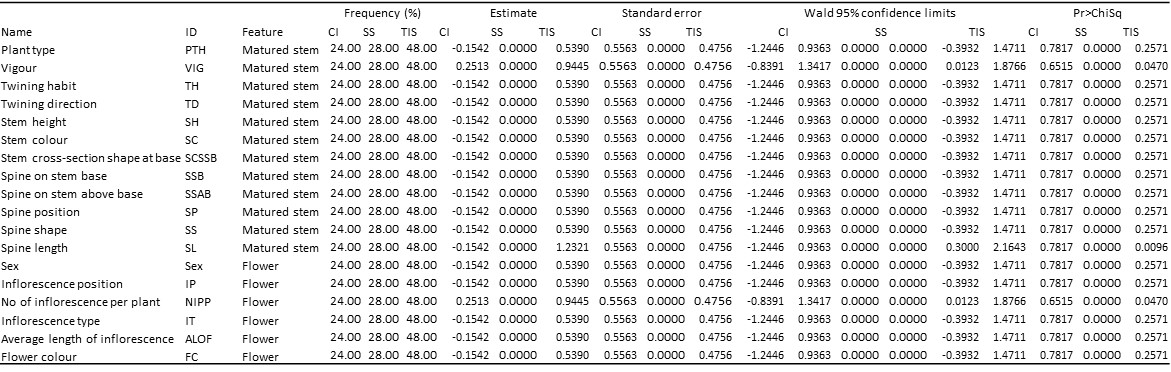
**

**S7d Table: Frequency table for Yam qualitative agro-morphological trait_ tuber**

**
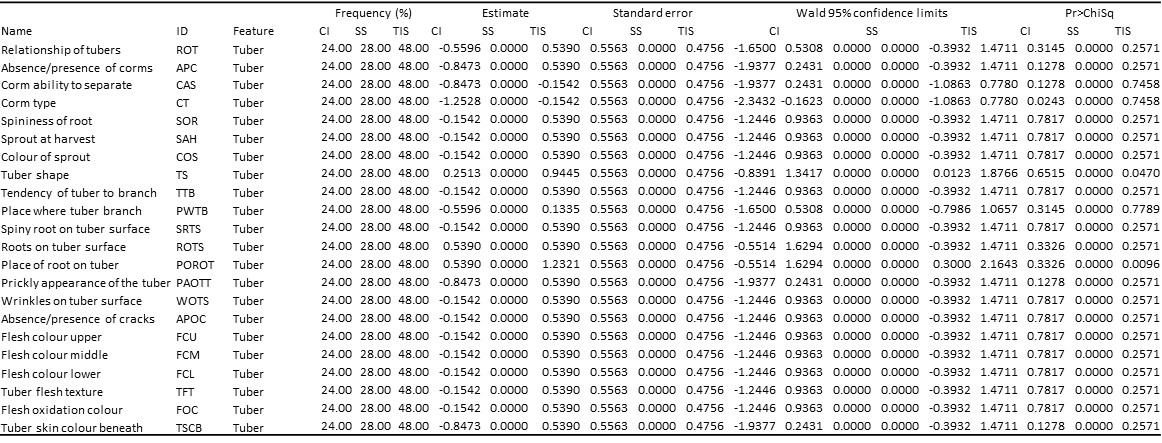
**

**S7e Table: ANOVA summary for yam quantitative traits**

| Source of Variation | df | Mean Square | | | | | | | | | | | |
| --- | --- | --- | --- | --- | --- | --- | --- | --- | --- | --- | --- | --- | --- |
|  |  | DOH | NTY1 | WTY1 | NTY2 | WTY2 | LOT | WOT | IL | DOFAE | MFL | NOSPP | NOI |
| System | 2 | 100.02ns | 1.30ns | 0.26ns | 8.48ns | 2.39ns | 16.39ns | 1.27ns | 13.33ns | 202.40ns | 0.10ns | 4.09ns | 12.21ns |
| Treatment | 3 | 79.21ns | 11.94ns | 1.22* | 19.13ns | 1.51ns | 17.97ns | 1.98ns | 12.00ns | 8011.99ns | 0.59ns | 3.79ns | 50.21ns |
| Treatment*System | 6 | 464.33ns | 17.29ns | 0.87* | 19.55ns | 5.52* | 72.90* | 0.86ns | 8.45ns | 12820.96ns | 0.46ns | 2.72ns | 34.03ns |
| Mean |  | 347.84 | 7.88 | 1.29 | 6.9 | 4.01 | 16.11 | 3.96 | 9.41 | 144.44 | 0.98 | 4.68 | 14.2 |
| Error |  | 262.64 | 10.72 | 0.26 | 8.76 | 1.39 | 20.19 | 1.35 | 6.99 | 14567.57 | 1.59 | 2.35 | 16.47 |
| CV |  | 4.65 | 41.55 | 39.77 | 42.87 | 29.42 | 27.89 | 29.27 | 28.09 | 83.56 | 127.95 | 32.75 | 28.58 |

|  | LSMean | | | | | | | | | |  |  |
| --- | --- | --- | --- | --- | --- | --- | --- | --- | --- | --- | --- | --- |
| System | DOH | NTY1 | WTY1 | NTY2 | WTY2 | LOT | WOT | IL | DOFAE | MFL | NOSPP | NOI |
| TIS | 349.15*** | 8.02*** | 1.16*** | 8.14*** | 4.50*** | 16.69*** | 4.05*** | 10.55*** | 151.17** | 0.93* | 4.73*** | 14.88*** |
| SS | 342.91*** | 7.20*** | 1.15*** | 5.75** | 3.37*** | 14.80*** | 3.54*** | 7.72*** | 148.41** | 0.53ns | 3.50*** | 12.45*** |
| CI | 350.87*** | 7.37*** | 1.53*** | 7.62*** | 4.66*** | 15.81*** | 3.70*** | 9.37*** | 140.12** | 1.06ns | 5.12*** | 15.00*** |

DOH, Date of harvest; NTY1, Number of tuber year 1; NTY2, Number of Tuber year 2; WTY1, Weight of tuber year 1; WTY2, Weight of tuber year 2; LOT, Length of tuber; WOT, width of tuber; IL, internode length, DOFAE, days to flowering after emergence; MFL, male flower length; NOSPP, Number of stem per plant; NOI, Number of internode, *, **, ***, p values significance at 0.05, 0.01, 0.001 respectively; ns, not significant; TIS, temporary immersion system; SS, Semi-Solid, CI, complete immersion
