# Supplementary material for: Agromorphologic, genetic and methylation profiling of Dioscorea and Musa species multiplied under three micropropagation systems
Source: PLoS One. 2019 May 16;14(5):e0216717. doi: 10.1371/journal.pone.0216717 (PMC6522119; doi:10.1371/journal.pone.0216717)
Supplement: S8 Table — (A) AMOVA summary for Musa under field condition using MSeI restriction enzyme, (B) AMOVA summary for Musa under in vitro conditions using MSeI restriction enzyme, (C) AMOVA summary for Dioscorea under in vitro condition using MSeI restriction enzyme (D) AMOVA summary for Dioscorea under field condition using MSeI restriction enzyme (E) AMOVA summary for Musa under field condition using HpaII restriction enzyme. Df, degree of freedom; SS, sum of squares; MS, mean squares; Est. var, estimated variance. (DOC) [file pone.0216717.s008.doc]

**S8 Table: (A) AMOVA summary for *Musa* under field condition using *MSe*I restriction enzyme, (B) AMOVA summary for *Musa* under *in vitro* conditions using *MSe*I restriction enzyme*,* (C) AMOVA summary for *Dioscorea* under *in vitro* condition using *MSe*I restriction enzyme (D) AMOVA summary for *Dioscorea* under field condition using *MSe*I restriction enzyme (E) AMOVA summary for *Musa* under field condition using *Hpa*II restriction enzyme**

| **(A)Summary AMOVA Table** | | | | | |
| --- | --- | --- | --- | --- | --- |
| **Source** | **df** | **SS** | **MSD** | **Est. Var.** | **%** |
| **Among Pops** | 2 | 842.6 | 421.3 | 7.821 | 3% |
| **Within Pops** | 44 | 13148.6 | 298.8 | 298.833 | 97% |
| **Total** | 46 | 13991.2 |  | 304.2 | 100% |
| **(B)Summary AMOVA Table** | | | | | |
| **Source** | **df** | **SS** | **MS** | **Est. Var.** | **%** |
| **Among Pops** | 2 | 380.667 | 190.333 | 0 | 0% |
| **Within Pops** | 6 | 1239.333 | 206.556 | 206.556 | 100% |
| **Total** | 8 | 1620 |  | 206.556 | 100% |
| **(C)Summary AMOVA Table** | | | | | |
| **Source** | **df** | **SS** | **MS** | **Est. Var.** | **%** |
| **Among Pops** | 2 | 613.833 | 306.917 | 34.153 | 17% |
| **Within Pops** | 9 | 1532.75 | 170.306 | 170.306 | 83% |
| **Total** | 11 | 2146.583 |  | 204.458 | 100% |
| **(D)Summary AMOVA Table** | | | | | |
| **Source** | **df** | **SS** | **MS** | **Est. Var.** | **%** |
| **Among Pops** | 2 | 385.494 | 192.747 | 0.374 | 0% |
| **Within Pops** | 22 | 4175.226 | 189.783 | 189.783 | 100% |
| **Total** | 24 | 4560.72 |  | 190.157 | 100% |
| **(E)Summary AMOVA Table** | | | | | |
| **Source** | **df** | **SS** | **MS** | **Est. Var.** | **%** |
| **Among Pops** | 2 | 445.184 | 222.592 | 2.175 | 1% |
| **Within Pops** | 44 | 8295.667 | 188.538 | 188.538 | 99% |
| **Total** | 46 | 8740.851 |  | 190.713 | 100% |

Df, degree of freedom; SS, sum of squares; MS, mean squares; Est. var, estimated variance
